# Comparison of Resting Full-Cycle Ratio and Fractional Flow Reserve in a German Real-World Cohort

**DOI:** 10.3389/fcvm.2021.744181

**Published:** 2021-12-24

**Authors:** Hendrik Wienemann, Annika Meyer, Victor Mauri, Till Baar, Matti Adam, Stephan Baldus, Marcel Halbach

**Affiliations:** ^1^University of Cologne, Faculty of Medicine and University Hospital Cologne, Clinic III for Internal Medicine, Cologne, Germany; ^2^Institute of Medical Statistics and Computational Biology, Faculty of Medicine, University of Cologne, Cologne, Germany

**Keywords:** coronary artery disease (CAD), fractional flow reserve (FFR), coronary physiology, invasive coronary angiography (ICA), resting full-cycle ratio (RFR)

## Abstract

**Objective:** The aim of this study was to evaluate non-hyperemic resting pressure ratios (NHPRs), especially the novel “resting full-cycle ratio” (RFR; lowest pressure distal to the stenosis/aortic pressure during the entire cardiac cycle), compared to the gold standard fractional flow reserve (FFR) in a “real-world” setting.

**Methods:** The study included patients undergoing coronary pressure wire studies at one German University Hospital. No patients were excluded based on any baseline or procedural characteristics, except for insufficient quality of traces. The diagnostic performance of four NHPRs vs. FFR ≤ 0.80 was tested. Morphological characteristics of stenoses were analyzed by quantitative coronary angiography.

**Results:** 617 patients with 712 coronary lesions were included. RFR showed a significant correlation with FFR (*r* = 0.766, *p* < 0.01). Diagnostic accuracy, sensitivity, specificity, positive predictive value, and negative predictive value of RFR were 78% (95% confidence interval = 75; 81), 72% (65; 78), 81% (77; 84), 63% (57; 69), and 86% (83; 89). Relevant predictors for discordance of RFR ≤ 0.89/FFR > 0.8 were LAD lesions, peripheral artery disease, age, female sex and non-focal stenoses. Predictors for discordance of RFR > 0.89/FFR ≤ 0.8 included non-LCX lesions, percent diameter stenosis and previous percutaneous coronary intervention in the target vessel. RFR and all other NHPRs were highly correlated with each other.

**Conclusion:** All NHPRs have a similar correlation with the gold standard FFR and may facilitate the acceptance and implementation of physiological assessments of lesion severity. However, we found ~20% discordant results between NHPRs and FFR in our “all-comers” German cohort.

## Introduction

Despite great advances in the prevention and treatment of cardiovascular diseases, ischemic heart disease remains one of the main causes of morbidity and mortality worldwide (1). Fractional flow reserve (FFR) is the gold standard pressure-derived index for the assessment of the physiological severity of coronary artery stenosis, and several guidelines and studies have highlighted the benefit of FFR-guided percutaneous coronary intervention (PCI) (2–6). FFR is derived from the ratio between the mean distal coronary artery pressure (Pd) to the mean aortic pressure (Pa) under maximum hyperemia and is considered to be significant with a threshold of ≤0.80 (7, 8). Administration of adenosine to achieve maximum hyperemia is associated with possible side effects (9), increased costs, and longer examination time, which may cause reservations against the application of FFR. Therefore, utilization remains low and heterogeneous between different hospitals in Germany (10).

The development of resting indices, referred to as non-hyperemic pressure ratio (NHPR), is therefore of great importance. Two large randomized clinical trials among patients with stable angina or acute coronary syndrome revealed that instantaneous wave-free ratio (iFR), which is calculated during the diastolic wave-free period and used by one of the leading manufacturers of pressure wires, is clinically non-inferior to FFR with regard to serious adverse events at one year (11, 12). Moreover, a previous study demonstrated that NHPRs have a comparable diagnostic quality for diagnosing positron emission tomography defined myocardial ischemia and show a comparable outcome with FFR at two years (13). Lately, it has been found that the resting full-cycle ratio (RFR), described as the lowest ratio of resting Pd/Pa during the entire cardiac cycle, which is used by another leading manufacturer, is diagnostically equivalent to iFR (13–15). Randomized trials comparing RFR and FFR are lacking. Although FFR ≤ 0.8 or NHPRs ≤ 0.89 can predict ischemia-inducing coronary stenoses with high accuracy, the correlation and agreement between FFR and NHPRs test results varies in clinical practice (13, 14, 16). Available data show that FFR and iFR test results are discordant in about 15–20% of cases, leading to uncertainty about revascularization decisions. This might be caused by limitations such as the assumption of maximal flow and minimal resistance occurring during the wave-free period of the diastole, which is the rationale of iFR (17, 18). Several clinical, angiographic, and hemodynamic factors contribute to iFR/FFR discordance (19–21). Available data do not represent a broader population in a real-world setting. Many patients were excluded from studies due to wide exclusion criteria such as vessels with a previous myocardial infarction, previous coronary artery bypass graft surgery, left main disease, chronic renal disease, bradycardia, atrial fibrillation or in-stent lesions (17, 22, 23). Furthermore, patients from Western Europe are underrepresented in most trials.

In the present retrospective, single-center study, we sought to investigate the diagnostic accuracy of FFR and NHPRs and the clinical utility of NHPRs and especially the relatively new non-hyperemic index RFR in a German “all-comers” population with intermediate coronary stenoses. The objective of this study is to assess the correlation of FFR and NHPRs in a real-world setting and evaluate predictors of discrepancies.

## Materials and Methods

### Study Population

From 9th of March 2015 until 15th of February 2019, a total of 696 adult patients underwent 869 pressure wire recordings of at least one intermediate coronary lesion (30–80%, determined visually by the treating physician) for standard clinical indications at the Heart Center of the University of Cologne. Pressure wire recordings were not performed in the following settings: (1) contraindication for adenosine, (2) cardiogenic shock (3), ST-segment elevation myocardial infarction, (4) culprit vessels in the setting of myocardial infarction (5), stenosis technically not suitable for analysis and (6) lesions without myocardial viability. All patients with FFR recordings in this period were included in this study, i.e., no patients were excluded from the analysis based on any baseline or procedural characteristics, except for insufficient quality of recorded traces, which impeded a reliable retrospective analysis (see pressure wire assessment). All collected patient data were anonymized before the analysis. The study design was approved by the local ethics committee and complied with the Declaration of Helsinki.

### Invasive Coronary Angiography and Quantitative Coronary Angiography

Coronary angiography was executed according to current guidelines and institutional standards by a femoral or radial approach, using a diagnostic or guiding catheter and low-osmolar contrast agents. Angiographic views were obtained in multiple standard projections. Diameter stenosis percentage, minimal and reference lumen diameter, and lesion length were assessed retrospectively by quantitative coronary angiography with validated software (CAAS II, Pie Medical System, Maastricht, The Netherlands).

### Pressure Wire Assessment

Interventional procedures and application of medication were performed according to current guidelines and manufacturer's and institutional standards. A pressure guidewire (Pressure Wire™ X Guidewire [Abbott Vascular Inc., Santa Clara, CA], or Verrata^®^ [Philips, San Diego, CA]) was calibrated, equalized, and advanced distal to the target lesion, and intracoronary nitrate was administered. Then continuous intravenous adenosine (140 μg/kg per min) was applied through a peripheral vein to induce hyperemia in the target vessel for FFR measurement. In the majority of cases, a pullback recording was made to exclude pressure drift.

**Figure 1:**
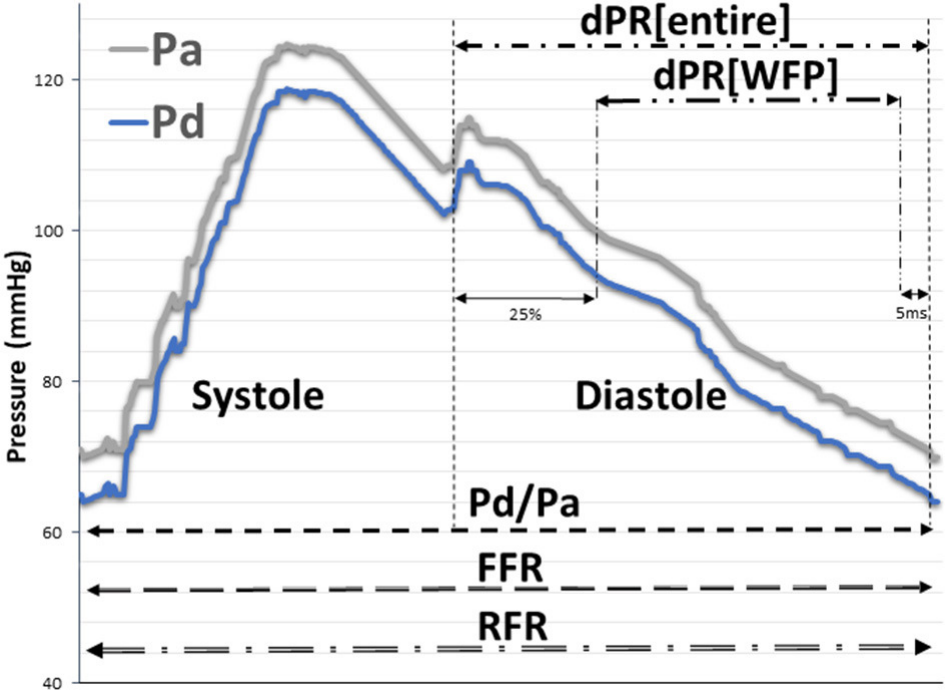
Schematic explanation of the NHPRs and FFR. Diastole starts at the nadir of the dicrotic notch. The dPR[entire] is defined as mean Pd/Pa over the entire diastole. The dPR[WFP] is defined as the mean Pd/Pa value in the wave-free period for 5 heart cycles (from 25% of the entire diastole to 5 ms before the end of diastole; equivalent to the definition of iFR). Whole cycle resting Pd/Pa was calculated continuously throughout the entire cardiac cycle for three heart cycles. FFR is defined as the lowest, artifact-free Pd/Pa during maximal hyperemia over at least three heart cycles (Pd/Pa and FFR are calculated in the same way, just under different conditions, i.e. resting conditions vs hyperemic conditions). RFR is defined as the lowest Pd/Pa value in systole and diastole (mean of 5 consecutive cardiac cycles). dPR[entire], diastolic pressure ratio during entire diastole; dPR[WFP], diastolic pressure ratio during wave-free period; FFR, fractional flow reserve; NHPR, non-hyperemic pressure ratio; Pa, aortic pressure; Pd, distal coronary pressure; RFR, resting full-cycle ratio.

For this study, all analyses were performed retrospectively using the raw data of pressure wire recordings. Distal (Pd) and aortic pressure (Pa) traces at baseline, i.e., before application of adenosine, were used to determine NHPRs. FFR was calculated using the lowest Pd/Pa under hyperemic conditions. FFR, RFR, diastolic pressure ratio during wave-free period (dPR[WFP]), diastolic pressure ratio during entire diastole (dPR[entire]), and Pd/Pa values (see Figure 1 for exact definitions) were calculated by a fully automated offline software algorithm at an independent core laboratory (CoroLab; Coroventis Research AB, Uppsala, Sweden). The thresholds for a hemodynamically significant stenosis (FFR ≤ 0.80, RFR ≤ 0.89, dPR[WFP] ≤ 0.89, dPR[entire] ≤ 0.89, and Pd/Pa ≤ 0.92) were defined according to current guidelines and recommendations. One hundred and fifty seven of 869 (18.1%) pressure recordings had to be excluded from the analysis, since resting or hyperemic periods could not be reliably identified or pressure recordings were instable. Hence, 617 patients with 712 stenoses were finally included in the study to assess the correlation of FFR to NHPRs. For patients with multivessel disease and recordings at different timepoints, baseline characteristics at first presentation were used for per-patient analysis.

In principle, assessment of non-focal (serial, diffuse) stenoses was performed using a dedicated software for calculation of quantitative flow ratio (QFR) based on coronary angiography (QAngio XA 3D version 2.0, Medis Medical Imaging Systems) (24).

### Statistical Analysis

Continuous variables are presented as mean ± standard deviation or median with interquartile range (IQR), while categorical variables are reported as frequencies and percentages. The differences were evaluated using Chi-square test or Fisher's exact test for categorical variables and Student's *t*-test or Mann-Whitney-*U* test for continuous variables, depending on their distribution. Kruskal–Wallis test was used to test for differences among >2 groups followed by *post-hoc* Mann-Whitney *U* tests with Bonferroni correction. Pearson's correlation coefficient was used to assess the relationship between several indices.

To assess the diagnostic value of NHPRs in comparison to FFR (≤0.80), the area under the curve (AUC) of the receiver-operating characteristic (ROC), as well as the accuracy metric were used. Diagnostic accuracy, sensitivity, specificity, negative, and positive predictive value, likelihood positive and negative ratio were calculated. Diagnostic agreement between the different indices was assessed by Bland-Altman plots with corresponding 95% limits of agreement.

Predictors of discordance between FFR and NHPRs were determined using a logistic regression model. The associated covariates with a *p*-value < 0.05 by forward selection in univariate analysis were included in the multivariable model after testing for multicollinearity. Backward stepwise selection was performed using a binary logistic model (backward elimination method: Wald). A two-tailed *p*-value of < 0.05 was regarded as statistically significant. Statistical analysis was conducted in SPSS Statistics, version 28 (IBM, Armonk, New York) and R programming language version 4.1.2 (R Foundation for Statistical Computing, Vienna, Austria).

## Results

### Patient and Vessel Characteristics

Clinical and angiographic characteristics are displayed in Table 1. The cohort consisted of patients with advanced age (median 69 years), with a high proportion of males (72.6%) and a high prevalence of comorbidities.

**Table 1:** Patient demographics and vessel characteristics.

	**All patients (n = 617)**
Age (years)	69 (61–77)
Female gender	169 (27.4)
Hypertension	448 (72.6)
Dyslipidemia	322 (52.2)
Diabetes mellitus	168 (27.2)
Insulin dependent	61 (9.9)
Current or past smoker	216 (35.0)
Peripheral arterial disease	47 (7.6)
Atrial fibrillation	65 (10.5)
Previous stroke	55 (8.9)
Previous myocardial infarction	180 (29.2)
Previous coronary artery bypass surgery	41 (6.6)
Family history of coronary artery disease	95 (15.4)
β-Blocker use	417 (67.6)
**Indication**	
NSTEMI	28 (4.5)
Unstable angina	137 (22.2)
Stable angina	192 (31.1)
Atypical angina	63 (10.2)
Silent ischemia	197 (31.9)
Multi-vessel disease	444 (72.0)
Previous percutaneous coronary intervention in the target vessel	126 (20.4)
	**All vessels (n=712)**
Left main	6 (0.8)
Left anterior descending artery	424 (59.6)
Ramus intermedius	11 (1.5)
Right coronary artery	143 (20.1)
Left circumflex artery	124 (17.4)
Coronary artery bypass	4 (0.6)
Non-focal stenoses	290 (40.7)
**Target segment**	
Proximal	308 (43.3)
Mid	300 (42.1)
Distal	104 (14.6)

*Data are given in n (%) or median (Q1–Q3)*.

The lesions were located most often in the left anterior descending coronary artery (LAD; 424 vessels; 59.6%). The reference diameter showed sufficient size for PCI (2.98 ± 0.57 mm). The lesions had a minimum diameter of 1.42 ± 0.42 mm, the mean percent diameter stenosis (%DS) was 52.9 ± 8.7 %, and the mean lesion length 15.6 ± 9.54 mm. Two hundred and ninety (40.7%) lesions were non-focal.

### Pressure Wire-Derived Indices

**Figure 2:**
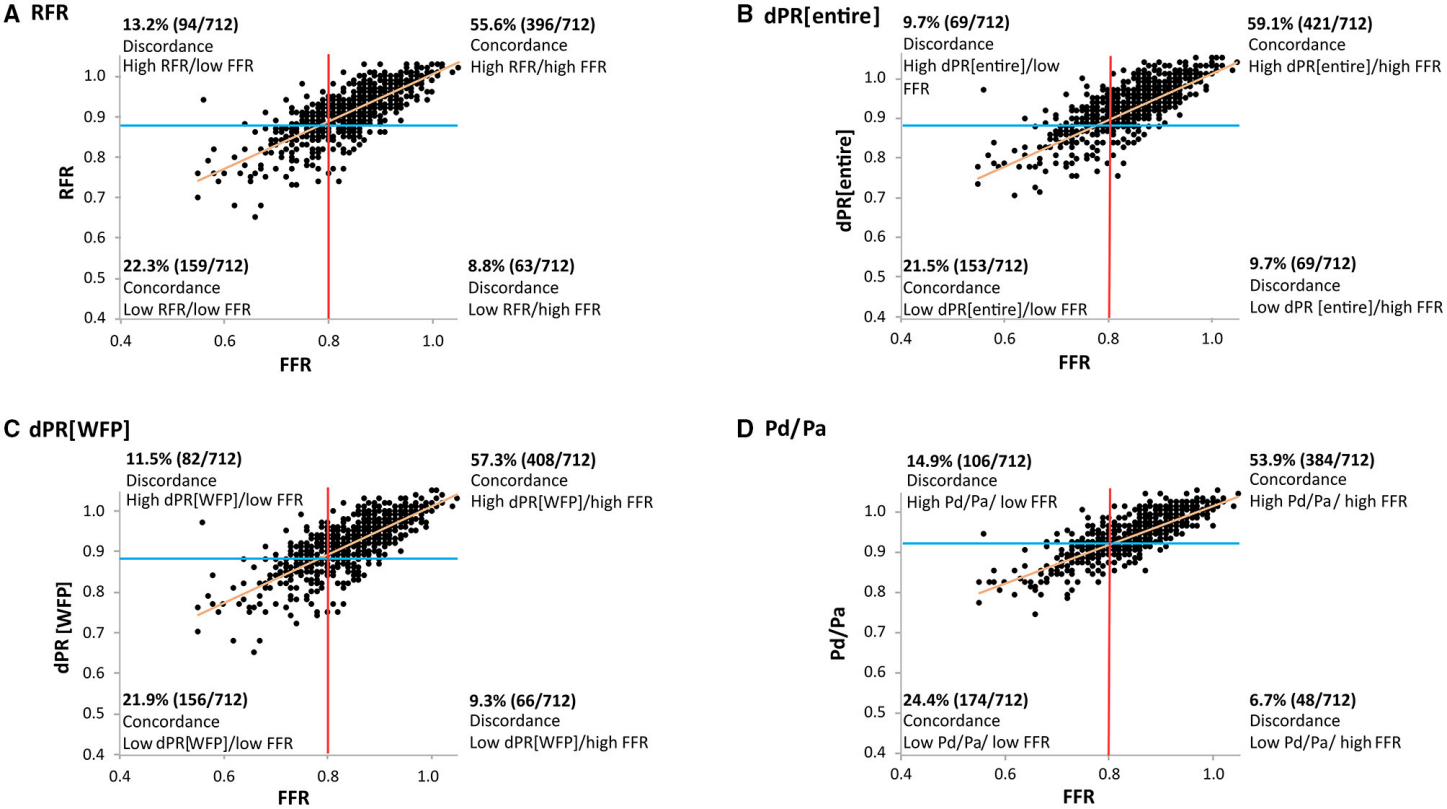
Concordance and discordance among NHPRs and FFR. **(A)** FFR and RFR showed a significant correlation (*r* = 0.766; *p* < 0.01), but 21.0% of lesions showed discordant classifications with FFR and RFR cutoff values of ≤0.80 and ≤0.89, respectively. **(B–D)** The correlation of FFR and dPR[entire], dPR[WFP] and Pd/Pa was also significant with a similar correlation coefficient. The frequency of discordant indices was comparable, too. dPR[entire], diastolic pressure ratio during entire diastole; dPR[WFP], diastolic pressure ratio during wave-free period; FFR, fractional flow reserve; NHPR, non-hyperemic pressure ratio; Pa, aortic pressure; Pd, distal coronary pressure; RFR, resting full-cycle ratio.

**Figure 3:**
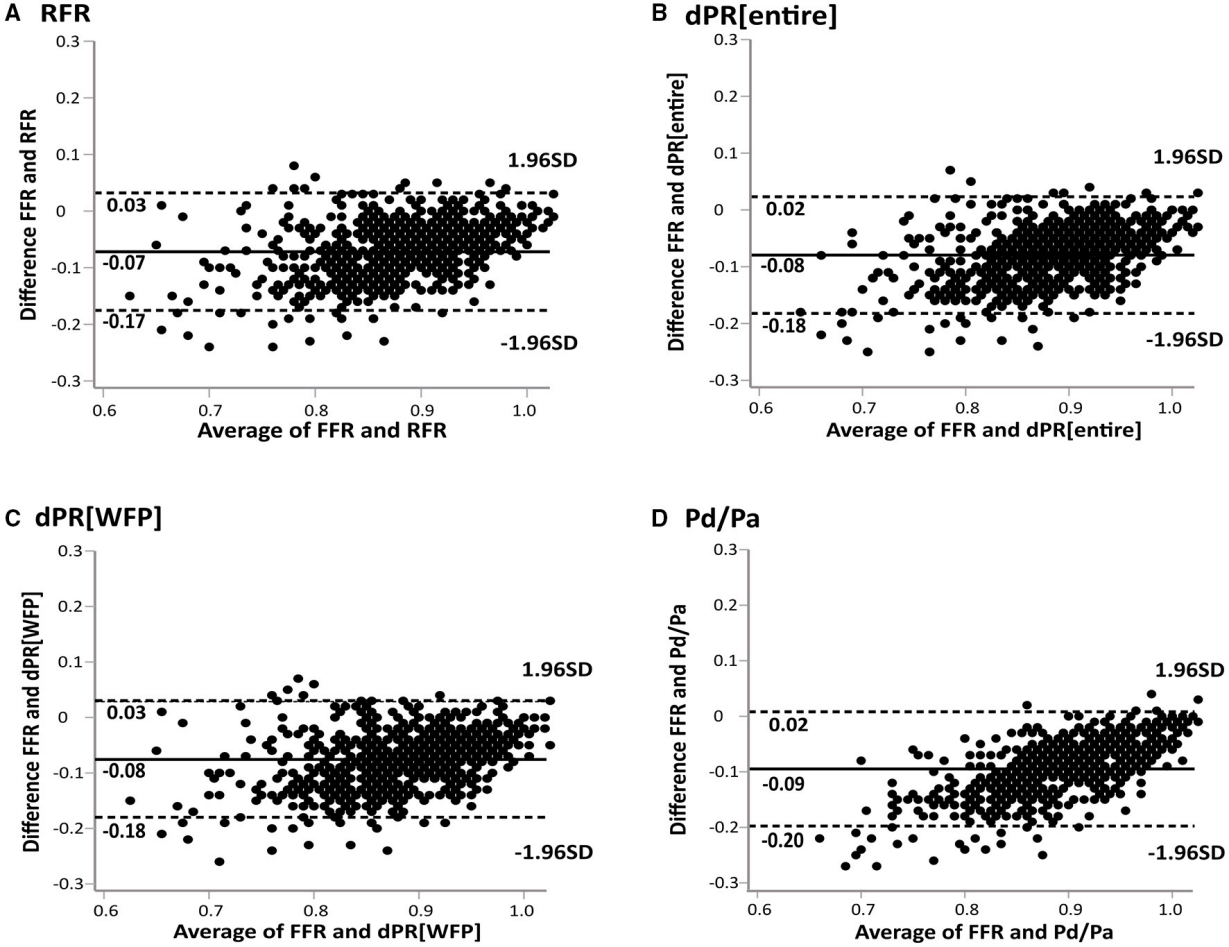
Bland-Altman plots of differences against the means are displayed for RFR **(A)**, dPR[entire] **(B)**, dPR[WFP] **(C)** and Pd/Pa **(D)**. Solid lines represent the mean bias, enclosed by the limits of agreement (dashed lines). The Bland-Altman plots demonstrate a good agreement between FFR and NHPRs. dPR[entire], diastolic pressure ratio during entire diastole; dPR[WFP], diastolic pressure ratio during wave-free period; FFR, fractional flow reserve; NHPR, non-hyperemic pressure ratio; Pa, aortic pressure; Pd, distal coronary pressure; RFR, resting full-cycle ratio.

The median values of pressure wire-derived indices were 0.84 (IQR: 0.79 to 0.90) for FFR, 0.91 (IQR: 0.88 to 0.96) for RFR, 0.92 (IQR: 0.88 to 0.96) for dPR[entire], 0.92 (IQR: 0.88 to 0.96) for dPR[WFP] and 0.94 (IQR: 0.90 to 0.97) for Pd/Pa, respectively. Ischemia defined as FFR ≤ 0.80 was detected in 222 of 712 lesions (31.2%; 201 of 617 patients). Nineteen (3%) minor side effects [chest discomfort/dyspnea (1.4%) and transient atrioventricular block (0.5%)] occurred during adenosine infusion, but no serious adverse events were observed. Resting indices suggested ischemia in 253 lesions (35.5%) for RFR, 222 (31.2%) for dPR[entire], 238 (33.4%) for dPR[WFP], and 280 (39.3%) for Pd/Pa. The prevalence of ischemia, regarding the cut-off value of 0.89, between dPR[WFP] and RFR were not statistically significant (*p* = 0.43). FFR was correlated with NHPRs (*r* = 0.766 for RFR; *r* = 0.763 for dPR[WFP]; *r* = 0.772 for dPR[entire]; *r* = 0.792 for Pd/Pa, *p* < 0.01; see Figure 2). Bland-Altman plot showed the mean bias ± SD between FFR and NHPRs (Figure 3).

**Figure 4:**
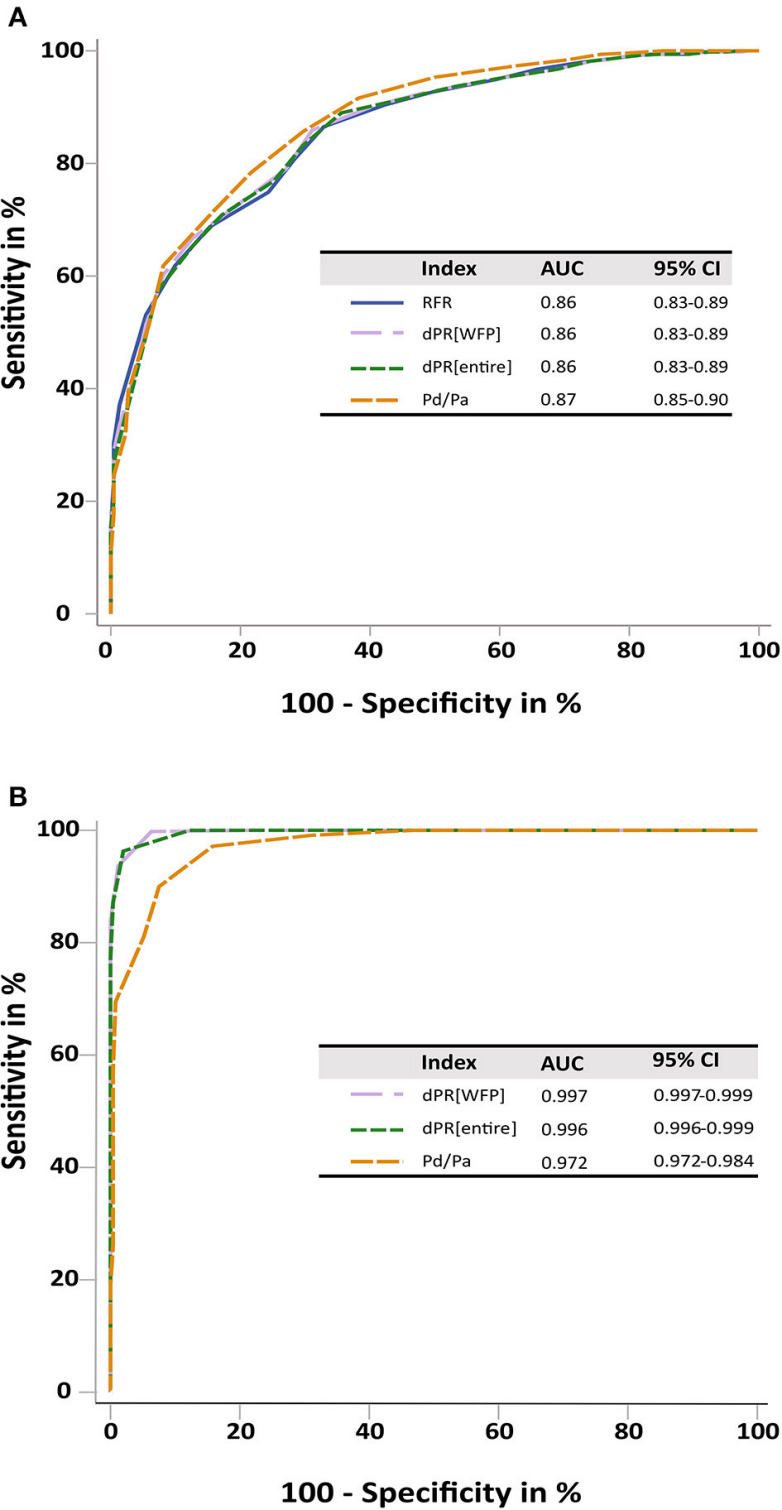
ROC curves. **(A)** ROC curves for RFR, dPR[entire], dPR[WFP] and Pd/Pa showed a similar performance of all NHPRs tested against an FFR ≤ 0.80. **(B)** ROC curves for dPR[WFP], dPR[entire], and Pd/Pa tested against RFR ≤ 0.89. All three indexes showed an excellent prediction for RFR defined ischemia, supporting the similar performance of all NHPRs. dPR[entire], diastolic pressure ratio during entire diastole; dPR[WFP], diastolic pressure ratio during wave-free period; FFR, fractional flow reserve; NHPR, non-hyperemic pressure ratio; Pa, aortic pressure; Pd, distal coronary pressure; RFR, resting full-cycle ratio.

The diagnostic performance of NHPRs to predict FFR ≤ 0.80 is shown in Table 2. RFR and the other NHPRs were highly correlated (*r* = 0.993 for dPR[WFP]; *r* = 0.992 for dPR[entire]; *r* = 0.943 for Pd/Pa, all *p* < 0.01). Increasing the per-lesion threshold to RFR ≤ 0.93 resulted in higher sensitivity of 90% to predict FFR ≤ 0.80, RFR ≤ 0.97 had 100% sensitivity. ROC curve analysis was performed to examine diagnostic performance of NHPRs using FFR ≤ 0.80 as the reference standard (Figure 4A). As shown in Figure 4B, large AUC were observed for the NHPRs using RFR ≤ 0.89 as the reference standard.

**Table 2:** Diagnostic performance of non-hyperemic pressure ratios for predicting FFR ≤ 0.80 at vessel level.

	**RFR ≤ 0.89**	**dPR[entire] ≤ 0.89**	**dPR[WFP] ≤ 0.89**	**Pd/Pa ≤ 0.92**
True positive, n	159 (22.3)	153 (21.5)	156 (21.9)	174 (24.4)
True negative, n	396 (55.6)	421 (59.1)	408 (57.3)	384 (53.9)
False positive, n	94 (13.2)	69 (9.7)	82 (11.5)	106 (14.9)
False negative, n	63 (8.8)	69 (9.7)	66 (9.3)	48 (6.7)
Accuracy, %	77.9 (74.7–80.9)	80.6 (77.5–83.5)	79.2 (76.0–82.1)	78.4 (75.2–81.3)
Sensitivity, %	71.6 (65.2–77.5)	68.9 (62.4–74.9)	70.3 (63.8–76.2)	78.4 (72.4–83.6)
Specificity, %	80.8 (77.0–84.2)	85.9 (82.5–88.9)	83.3 (79.7–86.5)	78.4 (74.5–81.9)
Positive predictive value, %	62.8 (56.6–68.8)	68.9 (62.4–74.9)	65.5 (59.1–71.6)	62.1 (56.2–67.8)
Negative predictive value, %	86.3 (82.8–89.3)	85.9 (82.5–88.9)	86.1 (82.6–89.1)	88.9 (85.5–91.7)
Positive likelihood ratio	3.7 (3.2–4.6)	4.9 (3.9–6.2)	4.2 (3.4–5.2)	3.6 (3.0–4.4)
Negative likelihood ratio	0.4 (0.3–0.4)	0.4 (0.3–0.4)	0.4 (0.3–0.4)	0.3 (0.2–0.4)

*Values are n (%) or n (95% confidence interval)*.

The analysis yielded a total recording of 3,560 cardiac cycles in 712 lesions. The RFR values showed a high reproducibility within the 5 measurements. The lowest ratio of resting Pd/Pa, i.e., the RFR, was located within systole in at least one cardiac cycle in 295 (8.2%) pressure tracings; in 80 (2.2%) pressure tracings the lowest ratio of Pd/Pa was located within systole in all analyzed cycles. The RFR was more frequently located within systole, when left circumflex artery (LCX) and right coronary artery (RCA) were examined [CABG 0%, RIM 0%, LAD 4.9% (404 out of 424), LCX 12.8% (108 out of 124), RCA 15.4% (121 out of 143), left main 16.7% (1 out of 6)].

### Discordance Between FFR and NHPRs

Concordant and discordant findings of FFR and NHPRs are summarized in Figure 2. One hundred and fifty seven lesions (22.1 %) in 138 patients (22.4%) showed discordant results of FFR and RFR, i.e., therapeutic decision would have differed. One hundred and forty eight lesions (20.8%) in 131 patients (21.2%) were discordant for FFR and dPR[WFP]. In the group with FFR ≤ 0.80 and RFR > 0.89, median FFR was 0.78 (IQR: 0.76–0.8) and median RFR 0.92 (0.91–0.93). In the group with FFR > 0.80 and RFR ≤ 0.89, median FFR was 0.84 (0.82–0.86) and median RFR 0.88 (0.85–0.89).

Patient and lesion characteristics of discordant and concordant RFR/FFR groups are shown in Tables 3A,B. In the univariate logistic regression analysis, age (*p* < 0.01), female sex (*p* = 0.01), peripheral artery disease (*p* = 0.01), LAD lesion (*p* < 0.01) und non-focal lesion (*p* = 0.02) were associated with discordance of RFR ≤ 0.89/FFR > 0.8 (Table 4A). Previous PCI in target vessel (*p* = 0.01), % DS (*p* < 0.01) and non-LCX lesions (*p* < 0.01) were associated with discordance of RFR > 0.89/FFR ≤ 0.8 (Table 4B). The multivariate analysis confirmed age (Odds Ratio [OR], 1.04; 95% confidence interval [CI], 1.02–1.07; *p* = 0.01), non-focal stenoses (OR, 1.84; 95% CI, 1.17–2.89; *p* = 0.01), female sex (OR, 1.70; 95% CI, 1.06–2.74; *p* = 0.03), peripheral artery disease (OR, 2.63; 95% CI, 1.36–5.09; p=0.01) and LAD lesion (OR, 3.22; 95% CI, 1.88–5.52; *p* < 0.01) as predictors of RFR ≤ 0.89/FFR > 0.8 (Table 4A). Previous PCI in target vessel (OR, 2.10; CI 1.15–3.85; *p* = 0.02), % DS (OR, 1.14; 95% CI, 1.1–1.19; *p* < 0.01) and non-LCX lesion (OR for LCX, 0.11; 95% CI, 0.02–0.52; *p* = 0.01) were confirmed as predictors of RFR > 0.89/FFR ≤ 0.8 in the multivariate analysis (Table 4B). The presence of acute coronary syndromes (ACS, mainly unstable angina) and the lesion location (proximal, medial or distal segment) did not predict discordance of RFR ≤ 0.89/FFR > 0.8 or RFR > 0.89/FFR ≤ 0.8 in the univariate analysis.

**Table 3A:** Comparison of patient characteristics between concordant and discordant cases of RFR and FFR.

	**RFR > 0.89** **FFR > 0.8** **Both negative**	**RFR ≤ 0.89** **FFR > 0.8** **iscordance**	**RFR ≤ 0.89** **FFR ≤ 0.8** **Both positive**	**RFR > 0.89** **FFR ≤ 0.8** **Discordance**	***p* value**	**Concordance**	**Discordance**	***p* value**
Age	69 (61–77)	74 (69–80)	68 (61–74)	65 (56–74)	**<** **0.01**	69 (61–79)	71 (63–78)	0.15
Female	104 (31.1)	31 (37.8)	25 (17.2)	9 (16.1)	**<** **0.01**	129 (26.9)	40 (29.0)	0.63
Diabetes mellitus	89 (26.6)	22 (26.8)	46 (31.7)	11 (19.6)	0.37	135 (28.2)	33 (23.9)	0.32
Insulin dependent	34 (10.2)	9 (11.0)	15 (10.3)	3 (5.4)	0.69	49 (10.2)	12 (8.7)	0.60
Dyslipidemia	172 (51.5)	46 (56.1)	70 (48.3)	34 (60.7)	0.38	242 (50.5)	80 (58.0)	0.12
Hypertension	241 (72.2)	59 (72.0)	104 (71.7)	44 (78.6)	0.78	345 (72.0)	103 (74.6)	0.54
Peripheral arterial disease	22 (6.6)	13 (15.9)	10 (6.9)	2 (3.6)	**0.02**	32 (6.7)	15 (10.9)	0.10
Current or former smoking	107 (32.0)	35 (42.7)	53 (36.6)	21 (37.5)	0.29	160 (33.4)	56 (40.6)	0.12
β-Blocker use	226 (68.1)	57 (70.4)	106 (73.1)	30 (53.6)	0.06	332 (69.6)	87 (63.5)	0.18
**Indication**								
NSTEMI	14 (4.2)	5 (6.1)	3 (2.1)	6 (10.7)	0.06	17 (3.5)	11 (8.0)	**0.03**
Unstable angina	69 (20.7)	17 (20.7)	41 (28.3)	10 (17.9)	0.23	110 (23.0)	27 (19.6)	0.40
Stable angina	98 (29.3)	24 (29.3)	49 (33.8)	21 (37.5)	0.54	147 (30.7)	45 (32.6)	0.67
Atypical angina	39 (11.7)	10 (12.2)	12 (8.3)	2 (3.6)	0.22	51 (10.6)	12 (8.7)	0.51
Silent ischemia	114 (34.1)	26 (31.7)	40 (27.6)	17 (30.4)	0.56	154 (32.2)	43 (31.2)	0.83

*Data are presented as n (%) or median (Q1–Q3). Bold numbers represent statistically significant p values*.

**Table 3B:** Vessel and lesion characteristics between concordant and discordant cases of RFR and FFR.

	**RFR > 0.89** **FFR > 0.8** **Both negative**	**RFR ≤ 0.89** **FFR > 0.8** **Discordance**	**RFR ≤ 0.89** **FFR ≤ 0.8** **Both positive**	**RFR > 0.89** **FFR ≤ 0.8** **Discordance**	***p* value**	**Concordance**	**Discordance**	***p* value**
No. of vessels	396 (55.7)	94 (13.2)	159 (22.3)	63 (8.8)		555 (78.0)	157 (22.0)	
**Target Vessel**								
Left anterior descending	174 (43.9)	75 (79.8)	131 (82.4)	44 (69.8)	**<** **0.01**	305 (55.0)	119 (75.8)	**<** **0.01**
Ramus intermedius	7 (1.8)	2 (2.1)	2 (1.3)	0 (0)	0.70	9 (1.6)	2 (1.3)	0.76
Right coronary artery	108 (27.3)	8 (8.5)	10 (6.3)	17 (27)	**<** **0.01**	118 (21.3)	25 (15.9)	0.14
Left circumflex artery	98 (24.7)	9 (9.6)	15 (9.4)	2 (3.2)	**<** **0.01**	113 (20.4)	11 (7)	**<** **0.01**
**Number of diseased vessels total**								
1	94 (23.7)	25 (26.6)	28 (17.6)	11 (17.5)	0.23	122 (22)	36 (22.9)	0.80
2	159 (40.2)	33 (35.1)	51 (32.1)	29 (46)	0.16	210 (37.8)	62 (39.5)	0.71
3	120 (30.3)	34 (36.2)	78 (49.1)	23 (36.5)	**<** **0.01**	198 (35.7)	57 (36.3)	0.88
**Lesion location**								
Proximal	191 (48.2)	39 (41.5)	58 (36.5)	20 (31.7)	**0.02**	249 (44.9)	59 (37.6)	0.11
Mid	147 (37.1)	47 (50.0)	74 (46.5)	32 (50.8)	**0.03**	221 (39.8)	78 (49.7)	**0.03**
Distal	58 (14.6)	8 (8.5)	27 (17)	11 (17.5)	0.27	85 (15.3)	19 (12.1)	0.31
Non-focal lesion	136 (34.3)	49 (52.1)	81 (50.9)	24 (38.1)	**<** **0.01**	217 (39.1)	73 (46.5)	0.10
**QCA analysis**								
Diameter stenosis, %	49.45 ± 7.26	51.69 ± 7.43	59.07 ± 8.1	61.04 ± 6.96	**<** **0.01**	52.2 ± 8.68	55.44 ± 8.56	**<** **0.01**
Reference diameter, mm	3.12 ± 0.61	2.9 ± 0.61	2.76 ± 0.57	2.79 ± 0.56	**<** **0.01**	3.02 ± 0.62	2.86 ± 0.59	**0.01**
Minimal lumen diameter, mm	1.58 ± 0.39	1.41 ± 0.39	1.13 ± 0.31	1.12 ± 0.32	**<** **0.01**	1.46 ± 0.42	1.3 ± 0.39	**<** **0.01**
Lesion length, mm	14.27 ± 8.53	14.61 ± 9.17	18.07 ± 10.96	19.46 ± 10.25	**<** **0.01**	15.36 ± 9.44	16.56 ± 9.88	0.17
**Pressure wire-derived index**								
FFR	0.89 (0.86–0.93)	0.84 (0.82–0.86)	0.75 (0.71–0.78)	0.78 (0.76–0.8)	**<** **0.01**	0.86 (0.79–0.91)	0.82 (0.79–0.85)	**<** **0.01**
RFR	0.95 (0.93–0.98)	0.88 (0.85–0.89)	0.85 (0.81–0.87)	0.92 (0.91–0.93)	**<** **0.01**	0.93 (0.88–0.97)	0.89 (0.87–0.91)	**<** **0.01**
dPR[WFP]	0.96 (0.93–0.98)	0.88 (0.86–0.89)	0.85 (0.81–0.87)	0.92 (0.91–0.94)	**<** **0.01**	0.94 (0.88–0.97)	0.89 (0.87–0.91)	**<** **0.01**
dPR[diastole]	0.96 (0.93–0.98)	0.88 (0.86–0.90)	0.86 (0.82–0.88)	0.92 (0.91–0.94)	**<** **0.01**	0.94 (0.89–0.98)	0.90 (0.88–0.92)	**<** **0.01**
Resting Pd/Pa	0.96 (0.95–0.99)	0.91 (0.9–0.92)	0.88 (0.85–0.9)	0.94 (0.92–0.95)	**<** **0.01**	0.95 (0.91–0.98)	0.92 (0.9–0.94)	**<** **0.01**

*Data are presented as n (%), mean SD, or median (Q1–Q3); Abbreviations as in the text. Bold numbers represent statistically significant p values*.

**Table 4A:** Independent predictors of disagreement between RFR and FFR.

	**Discordance with RFR** **≤0.89 and FFR** **>0.8**					
	**Univariate Analysis**			**Multivariate Analysis**		
	**Odds ratio**	**95% CI**	***P*-value**	**Odds ratio**	**95% CI**	***P*-value**
Age, per 1-year increment	1.04	1.02–1.07	**<** **0.01**	1.04	1.02–1.07	**0.01**
Female	1.86	1.19–2.92	**0.01**	1.70	1.06-2.74	**0.03**
Diabetes mellitus	0.99	0.6–1.61	0.96			
Hypertension	1.06	0.65–1.74	0.80			
Hyperlipidemia	1.24	0.8–1.92	0.33			
Current or former smoking	1.21	0.77–1.89	0.41			
Peripheral arterial disease	2.42	1.29–4.54	**0.01**	2.71	1.39–5.28	**0.01**
NSTEMI or unstable AP	0.97	0.59–1.59	0.91			
Previous PCI in target vessel	1.33	0.81–2.19	0.26			
Previous myocardial infarction	1.12	0.70–1.80	0.64			
Non-focal lesion	1.70	1.10–2.63	**0.02**	1.84	1.17–2.89	**0.01**
Multivessel disease	0.85	0.53–1.38	0.52			
Proximal or mid segment^*^	1.98	0.93–4.21	0.08			
Proximal segment	0.93	0.60–1.44	0.73			
Mid segment	1.38	0.90–2.14	0.14			
Distal segment	0.51	0.24–1.08	0.08			
% DS	0.98	0.96–1.01	0.14			
Minimal lumen diameter	0.96	0.58–1.62	0.89			
Lesion length	0.99	0.96–1.01	0.27			
Lesion location LAD	3.04	1.8–5.16	**<** **0.01**	3.22	1.88–5.52	**<** **0.01**
Lesion location RCA	0.33	0.16–0.7	**<** **0.01**			
Lesion location LCX	0.46	0.23–0.95	**0.04**			

* *Target lesion located in the proximal or mid segment of the target vessel. CI, confidence interval; other abbreviations as in the text. Bold numbers represent statistically significant p values*.

**Table 4B:** Independent predictors of disagreement between RFR and FFR.

	**Discordance with RFR** **>0.89 and FFR≤** **0.8**					
	**Univariate Analysis**			**Multivariate Analysis**		
	**Odds ratio**	**95% CI**	***P*-value**	**Odds ratio**	**95% CI**	***P*-value**
Age, per 1-yr increment	0.97	0.95–1.00	**0.02**			
Female	0.50	0.26–0.99	**0.05**			
Diabetes mellitus	0.62	0.32–1.19	0.15			
Hypertension	1.37	0.74–2.55	0.31			
Hyperlipidemia	1.21	0.72–2.04	0.47			
Current or former smoking	1.10	0.64–1.88	0.73			
Peripheral arterial disease	0.33	0.08–1.4	0.13			
NSTEMI or unstable AP	1.38	0.80–2.41	0.25			
Previous PCI in target vessel	2.04	1.18–3.55	**0.01**	2.10	1.15–3.85	**0.02**
Previous myocardial infarction	0.99	0.56–1.75	0.97			
Non-focal lesion	0.89	0.52–1.51	0.87			
Multivessel disease	1.73	0.88–3.4	0.11			
Proximal or mid segment^*^	0.79	0.4–1.57	0.50			
Proximal segment	0.56	0.34–1.02	0.06			
Mid segment	1.48	0.89–2.48	0.14			
Distal segment	1.27	0.64–2.51	0.53			
% DS	1.14	1.1–1.18	**<** **0.01**	1.14	1.10–1.19	**<** **0.01**
Minimal lumen diameter	0.09	0.04–0.2	**<** **0.01**			
Lesion length	1.04	1.02–1.06	**<** **0.01**			
Lesion location LAD	1.64	0.94–2.87	0.08			
Lesion location RCA	1.53	0.85–2.77	0.16			
Lesion location LCX	0.14	0.03–0.59	**0.01**	0.11	0.02–0.52	**0.01**

* *Target lesion located in the proximal or mid segment of the target vessel. CI confidence interval; other abbreviations as in the text. Bold numbers represent statistically significant p values*.

## Discussion

We validated NHPRs, with a special focus on RFR, vs. the gold standard FFR in patients with angiographically intermediate coronary stenoses in a German “all-comers” population. All NHPRs correlated very well with each other and showed a diagnostic accuracy of 77–81% for FFR. Discrepancies between FFR and RFR were found in 22.0% of recordings, and a higher rate of ischemia was diagnosed by RFR compared to FFR. We identified several clinical parameters that may predict discordance of RFR and FFR.

FFR-guided revascularization is supported by several randomized trials (3–5) and recommended by European guidelines (2, 6). Despite convincing evidence and clear recommendations, the rate of FFR-guided revascularizations is low (e.g., ~17% of interventions performed in Germany in 2019 were FFR-guided; unpublished survey of the German Society of Cardiology 2019, DGK). Reasons may include the costs and prolongation of procedures associated with administration of adenosine. Adenosine-free NHPRs may facilitate the acceptance and implementation of physiological assessments. Two randomized trials have demonstrated that iFR-guided treatment is non-inferior to FFR-guided treatment (11, 12), while other NHPRs were not yet validated in randomized trials. However, discrepancies between NHPRs and FFR have been reported, and literature about this topic is growing (17, 20, 25), but still limited by a small number of examined patients (26).

Our study represents a broad “all-comers” German population, which is one of the largest studied so far. Previous studies on NHPRs mostly enrolled Asian, American or Scandinavic patients, which may not be fully representative of the Central European population due to different patient characteristics and regional differences in the use of percutaneous coronary interventions (13–15). Furthermore, 26.7% of the patients presented with ACS in our study, which is more than in previous studies (23).

Diagnostic accuracy of NHPRs was between 78–81%, if the “gold standard” FFR is taken as reference, which must be rated as only moderate. This finding is in line with most prior studies (14, 16, 23). A slightly higher diagnostic accuracy of 86.6% for RFR and 87.5% for dPR[entire] was reported in the analysis of the 3V FFR-FRIENDS study (13), which included a population with less severe stenoses as indicated by a median FFR of 0.89 as compared to 0.84 in the present study. The moderate correlation of NHPRs and FFR justifies a carful interpretation of resting indices, e.g., adding FFR recordings in cases of borderline NHPRs may be considered, but it does not question the high value of resting indices, since the feasibility and costs are superior to FFR, which will facilitate the broad application of pressure-wire recordings and help to avoid clearly inferior angiography-based decision making.

To our knowledge, this is the largest study focusing on discordant findings of RFR and FFR. Discordant results were found in 22% of the lesions, which is more frequent compared to previous smaller studies with a range from 7.2–19.7% (14, 22, 26–28). This may be attributed to the broad inclusion criteria, which may affect physiologic measurements and cause discrepancies (21). Discordant findings of NHPRs and FFR have been associated with worse prognosis as compared to concordant negative indices in previous trials (28). On the other hand, iFR-based revascularization was non-inferior to an FFR-based approach despite lower revascularization rates in both randomized trials (11, 12). There is an ongoing debate, whether discordant lesions should be revascularized (20, 27, 28). Thus, there is a strong need for more data on the clinical impact of discordance on outcome as well as the indication for revascularization, which was beyond the scope of our study and encourages future - ideally randomized prospective—trials.

Since a single index is usually applied in clinical practice and determination of both resting and hyperemic indices is performed only in the minority of cases, discordance of results is usually inapparent, which increases the importance of potential predictors of discordant findings. Several clinical factors such as gender, anemia, LV diastolic dysfunction, diabetes mellitus and angiographic factors have been proposed as predictors of discordance of low NHPRs /high FFR (19, 20, 22, 26, 29), i.e., revascularization would be performed based on an NHPRs but deferred based on FFR. In our population, female sex, age, LAD lesion, PAD and non-focal lesion were predictors of RFR ≤ 0.89/FFR > 0.8.

Multivariate analysis revealed LAD lesions as a highly relevant predictor for RFR ≤ 0.89/FFR > 0.8 with an odds ratio of 3.17, confirming the findings of present studies of NHPRs (26, 29).

Kobayashi et al. speculated that the larger myocardial territory supplied by LM/LAD vs. non-LAD may cause larger coronary flow variation between resting and hyperemic conditions, which could be responsible for the difference (25).

PAD was a relevant predictor of discrepancy of RFR ≤ 0.89 /FFR > 0.8 with an odds ratio of 2.63. The same finding was reported by Goto et al. (26). Pellegrino et al. reported that the coronary flow reserve (CFR) was significantly lower in patients with PAD than in those without PAD (30), which could explain the discordance, since reduced flow may lead to an underestimation of stenosis severity by FFR. Also Cook et al. suggested that iFR ≤ 0.89 /FFR > 0.8 might be explained by differences in hyperemic coronary flow (17).

The odds ratio was 1.67 for female sex in the multivariate analysis. Two previous studies reported that female sex was significantly associated with NHPRs ≤ 0.89/FFR > 0.8 (20, 22), but not all studies confirmed this finding (26). A *post-hoc* analysis of the DEFINE-FLAIR study demonstrated that an FFR-guided strategy was associated with a higher rate of revascularization than an iFR-guided strategy in men, but not in women (31), so gender differences of pressure-derived indices appear to have a clinical impact. Kobayashi et al. speculated that women tend to have a higher coronary flow at rest leading to higher trans-stenotic pressure losses and lower NHPRs (32).

The odds ratio for age was only 1.04, so despite statistical significance in the multivariate analysis, the impact of age appears to be of minor relevance. Age was also not found as a predictor of discordance in most previous studies for FFR and NHPRs (19, 20, 22, 26). Just like described above for PAD, older age is associated with a decrease in CFR (33) and an increase in microvascular resistance under hyperemia, which may cause an underestimation of stenosis severity by FFR.

The presence of non-focal stenoses, i.e., serial stenoses or diffuse disease, was another predictor for discordance of RFR ≤ 0.89/FFR > 0.8. It has been described that the presence of serial stenoses increases the risk of discordance of FFR and iFR (34). In non-focal disease, downstream stenoses may impede hyperemic flow, thus FFR may underestimate the true physiological impact of analyzed stenoses. Therefore, NHPRs may be superior to FFR in the assessment of non-focal stenoses, although this hypothesis needs confirmation by further clinical trials.

Interestingly, RFR suggested ischemia more frequently than FFR (35.5% of patients had RFR ≤ 0.89, 31.2% had FFR ≤ 0.80) in the present study, although most previous studies reported slightly higher rates of ischemia determination by FFR (22, 26). Both randomized studies of iFR- vs. FFR-guided revascularization demonstrated a lower number of revascularizations in the iFR group (12, 35).

Predictors of RFR > 0.89 /FFR ≤ 0.8 discordance, i.e., revascularization would be performed based on FFR but deferred based on an NHPR, were %DS, history of percutaneous coronary intervention in the target vessel, and non-LCX lesions in the multivariate analysis. The relevance of %DS was small with an odds ratio of 1.14. %DS was also a predictor of iFR > 0.90 /FFR ≤ 0.8 discordance in the work by Lee et al. (19). Other studies could not predict this type of discordance, maybe due to the small number of analyzed lesions (22, 26). History of percutaneous coronary intervention in the target vessel (odds ratio 2.10) and non-LCX lesions (odds ratio 0.11 for LCX) were stronger predictors of RFR > 0.89 /FFR ≤ 0.8 discordance in the present trial.

Lesion location (proximal, medial and distal segments) and multivessel disease had no significant effect on the concordance or discordance of indices. Due to the relatively high number of patients with ACS in our study, we could analyze the interaction between the presence of an ACS and the concordance or discordance of indices, which was not significant. This is an interesting finding, since microvascular function may be altered in patients with ACS, which may diminish the effect of adenosine and lead to false negative FFR values (36). Moreover, resting coronary flow in non-culprit ACS lesions may be higher than in stable coronary artery disease (37), which may also contribute to discordance of indices. Our finding of no relevant interaction between the presence of an ACS and the discordance of RFR and FFR is in line with a previous study on iFR and FFR (38).

A subgroup analysis of patients with and without prior myocardial infarction (irrespective of the location) revealed no interaction with the discordance of indices, too. However, we cannot exclude that an analysis of prior myocardial infarction in the territory of the examined vessel may have led to different findings. Unfortunately, information on the exact location of prior myocardial infarction was not available for our population.

It was speculated that coronary stenoses with iFR > 0.90 /FFR ≤ 0.75 show similar coronary flow properties as angiographically unobstructed vessels and, in cases with normal or high CFR and FFR ≤ 0.75, the low FFR may reflect high flow states in response to adenosine rather than significant stenoses, which would be associated with a good prognosis (39, 40). In line with this hypothesis, the randomized trials DEFINE-FLAIR and iFR-SWEDEHEART showed that the risk of coronary events was not increased, although more lesions were deferred for revascularization based on iFR compared to FFR (41).

In our study, all parameters of diagnostic performance were similar for all analyzed NHPRs, increasing the body of evidence that NHPRs are largely comparable among one another, as suggested by one previous trial (13). Thus, a special algorithm of any company has little advantage compared to the “open source” index Pd/Pa. One potential advantage may be that Pd/Pa shows a higher susceptibility to pressure-sensor drifts and to pressure-curves artifacts (42).

Since iFR is the only NHPR with evidence from randomized controlled trials, differences between iFR and other NHPRs are of particular interest. Van't Veer et al. reported that available diastolic resting indices calculated in the “wave-free period” are identical to iFR despite minor differences in algorithms (43), so we consider dPR[WFP] used in our study to be equal to iFR. We found a difference of only 2.2% for dPR[WFP] vs. RFR in the rate of ischemia determination, which was not statistically significant. So, an RFR-based approach would have led to the same treatment strategy as an iFR-based in the majority of cases.

Accordingly, only 2.2% of RFR measurements detected the lowest Pd/Pa in systole. This is much lower than reported in most previous studies with a range from 11.4–12.2% (13, 14). Flow profiles between LCA und RCA are different (44), which may be explained by differences in supplied myocardial mass (45) and may have an impact on the timing of lowest Pd/Pa. In the VALIDATE study, lowest Pd/Pa was detected in systole in 32.4% in the RCA (14). In the present study, lowest Pd/Pa was in systole in 15.4% of RCA analyses. In contrast, Hoshino et al. found only 2.4% of RFR values in systole in the RCA (23).

### Limitations

Our trial has several limitations. It was an observational retrospective cohort study conducted at a single center. Revascularization of the target lesion was based on FFR values and operators' decision, and not on NHPRs. Patient selection for pressure-wire assessment was also within the discretion of the treating physician, which may have led to bias. However, we intended to study pressure wire-derived indices under real-life conditions. Another limitation is that we did not investigate clinical outcomes after revascularization. Thus, the clinical impact of discrepancies between FFR and NHPRs could not be assessed.

Our cohort comprised patients with diabetes, CABG, and intake of beta-blockers. On the other side, we consider the broad “all-comers” population as one strength of the present study, since it probably better reflects the realities of care than previous studies with more extensive exclusion criteria.

No assessment of microvascular function or coronary flow was performed. A better understanding of microcirculation and coronary flow under baseline and hyperemic conditions would have improved the interpretation of discrepant findings of NHPRs and FFR.

A total of 157 out of 869 (18.1%) pressure tracings could not be analyzed. Previous studies have shown that even in the context of a prospective clinical trial, physiological assessments of stenosis severity may be limited by pressure tracings being unanalyzable due to artifacts and failure in up to 30% (16, 25). Patients were not excluded from our analysis based on any clinical parameters, but all non-analyzed recordings had to be excluded due to insufficient tracings (e.g., dampened aortic pressure, no stable resting or hyperemic recording, resting or hyperemic period not recorded at all). We did not collect clinical data of these patients, but we consider it very likely, that the technical shortcomings leading to exclusion of tracings from the analysis were not associated with any clinical characteristics.

The external analysis of pressure curves by an expert core lab is not fully representative for the real-life situation, however it reduces inter-observer variability and ensures reliability of the calculations. Moreover, due to the retrospective nature of our study and the incapability of commercially available systems to calculate all investigated resting indices online in the catheterization laboratory, an offline analysis was necessary.

## Conclusions

All NHPRs have a similar correlation with the gold standard FFR and may facilitate the acceptance and implementation of physiological assessments of lesion severity. However, we found ~20% discordant results between NHPRs and FFR in our “all-comers” German cohort. Most relevant predictors for discordance of RFR ≤ 0.89 /FFR > 0.8 were LAD lesions, PAD, female sex and non-focal stenoses. Strong predictors for discordance of RFR > 0.89/FFR ≤ 0.8 were non-LCX lesions and previous percutaneous coronary intervention in the target vessel. The impact of discrepant findings on outcome and the optimal treatment strategy needs to be further elucidated by future prospective trials.

## Data Availability Statement

The raw data supporting the conclusions of this article will be made available by the authors, without undue reservation.

## Ethics Statement

The studies involving human participants were reviewed and approved by Ethics Committee Medical Faculty, University of Cologne. The Ethics Committee waived the requirement of written informed consent for participation.

## Author Contributions

HW, MH, and SB: study concept and design. HW, AM, VM, and TB: acquisition and analysis or interpretation of data. HW: writing and original draft preparation of the manuscript. HW, VM, MA, SB, and MH: discussion or critical revision of the interpretation for important intellectual content. MH: study supervision. All authors reviewed the manuscript.

## Funding

The corelab analysis at Coroventis Research AB was supported by Abbott Vascular.

## Conflict of Interest

SB and MH received institutional grant support and speakers' honoraria from Abbott Vascular. HW received institutional grant support from Abbott Vascular. Abbott Vascular had no influence on the design of the study, data analysis or interpretation of findings. The remaining authors declare that the research was conducted in the absence of any commercial or financial relationships that could be construed as a potential conflict of interest.

## Publisher's Note

All claims expressed in this article are solely those of the authors and do not necessarily represent those of their affiliated organizations, or those of the publisher, the editors and the reviewers. Any product that may be evaluated in this article, or claim that may be made by its manufacturer, is not guaranteed or endorsed by the publisher.

## Abbreviations

%DS: percent diameter stenosis
CABG: coronary artery bypass surgery
CFR: coronary flow reserve
dPR[entire]: diastolic pressure ratio during entire diastole
dPR[WFP]: diastolic pressure ratio during wave-free period
FFR: fractional flow reserve
LAD: left anterior descending
LM: left main coronary artery
LCX: left circumflex artery
NHPRs: non-hyperemic pressure ratios
Pa: proximal aortic pressure
Pd: distal arterial pressure
PAD: peripheral artery disease
PCI: percutaneous coronary intervention
RCA: right coronary artery
RFR: resting full-cycle ratio
RIM: Ramus intermedius
QCA: quantitative coronary angiography
QFR: quantitative flow ratio.
